# Drug supply and assurance: a cross-sectional study of drug shortage monitoring varieties in China

**DOI:** 10.1186/s12889-024-19361-5

**Published:** 2024-07-30

**Authors:** Yipeng Lan, Xiaofeng Lin, Qiannan Chen, Li Wang, Lihua Sun, Zhe Huang

**Affiliations:** 1 School of Business Administration, Shenyang Pharmaceutical University, Shenyang, China; 2 Institute of Drug Regulatory Science, Shenyang Pharmaceutical University, Shenyang, China

**Keywords:** Drug shortages, Monitored varieties, Drug supply and assurance, Essential medicines, Cross-sectional study, China

## Abstract

**Background:**

Drug shortage is a worldwide problem that seriously threatens public health. China released the most comprehensive list of key drug shortage monitoring varieties ever in 2022. We aim to analyze the attributes and characteristics of the medicines within the list to provide a reference for improving China’s supply security of shortage drugs.

**Methods:**

We used public data to extract information on drug types, dosage forms, indications, classification of clinical uses, whether they were included in medical catalogs such as the National Essential Drugs, and the number of drug and active pharmaceutical ingredient (API) manufacturers. A descriptive statistical analysis was used.

**Results:**

Of the 980 drugs on the list, 99.59% were chemicals and 92.65% were injectables. Drugs for blood and hematopoietic organs, the cardiovascular system, and the digestive tract and metabolism ranked among the top three shortages. Verification of the medical catalogs showed that 90.41% of the drugs belonged to the national essential drugs, 95.10% were medicare drugs, 2.55% were volume-based procurement drugs, and 14.70% were for rare diseases, and 42.04% were for children. In terms of drug supply capacity, 21.33% of drug approvals are less than 10, and there were even 26 drugs for exclusive production, close to 90% of manufacturers need to purchase APIs from outside. Among the 256 APIs included in the list, 152 APIs had less than 10 manufacturers, and there were even 5 APIs produced by only one enterprise nationwide.

**Conclusions:**

The situation of drug shortages in China was severe and complex, with serious shortages of medicines adapted to basic medical and healthcare needs and clinically necessary medicines, and a need to improve the production capacity of drugs and the ability to supply APIs. We recommend strengthening drug monitoring and stockpiling and accelerating the approval of shortage drugs to improve drug supply security.

**Supplementary Information:**

The online version contains supplementary material available at 10.1186/s12889-024-19361-5.

## Background

As early as 2006, the World Health Organization (WHO) identified drug shortage as a worldwide problem [1]. Both developed and developing countries are facing drug shortages, which pose a threat to the health and quality of life of citizens. Currently, there is a lack of a uniform definition of shortage drugs globally, and countries have different criteria for inclusion and statistics of shortage medicines [2]. In the United States, there were 49 new shortage drugs in 2022, and the Food and Drug Administration (FDA) has stated that drug shortages continue to be a top priority for the FDA because they can pose a significant public health threat [3]. In addition, it has been reported that the number of drug shortages in the European Union increased 20-fold between 2000 and 2018 [4]. Today, drug shortages in European countries remain a serious problem [5, 6]. In France, drug shortage reports increased from 404 in 2013 to 3,761 in 2022, a nearly tenfold increase in ten years [7]. Another survey showed that more than 83% of UK pharmacists experienced drug shortages three or more times per week, and in some cases could not even bring in much-needed patient care [8, 9]. Between April 2017 and April 2020, 13,329 medicines were at risk of shortages in Canada, with 44.7% of them experiencing at least 1 shortage in the past 5 years [10]. In developing countries, Pakistan has also experienced shortages of several antineoplastic and anesthetic drugs since 2021, severely affecting patient care and survival [11, 12].

In China, drug shortages are equally serious. According to the definition of the National Health Commission (NHC), shortage drugs are drugs approved for marketing by the Chinese drug administration authorities, that are clinically necessary and irreplaceable or incompletely replaceable and are in insufficient or unstable supply at a certain period or in a certain region [13]. In recent years, there have been many instances of stock-outs of cheap, life-saving medicines in China, such as protamine (medicine for rescuing coagulation disorders), brompheniramine tablets (special medicine for myasthenia gravis), and pingyangmycin (commonly used in children’s oncology), as well as phased shortages of supply of some small varieties and batches of medicines (e.g., medicines for rare diseases and cancers), and of some emergency medicine, and medicines for women and children [14, 15]. A study of Shaanxi Province, China, concluded that in 2016, there were shortages in the supply of 8 traditional Chinese medicines and 87 biological products and chemicals in the province [16]. In addition, between 2018 and 2021, 24 provinces in China announced a total of 408 drug shortages [17]. Drug shortage has multiple negative impacts on healthcare: (i) it may delay or fail to provide needed care to patients, resulting in potential failures in medical care [18]; (ii) it may force healthcare professionals to use second-line alternative medications, which are not only less effective but also potentially risky [19, 20]; (iii) shortages of some life-saving or emergency medications can delay patient care and even threaten lives [21, 22].

**Fig. 1:**
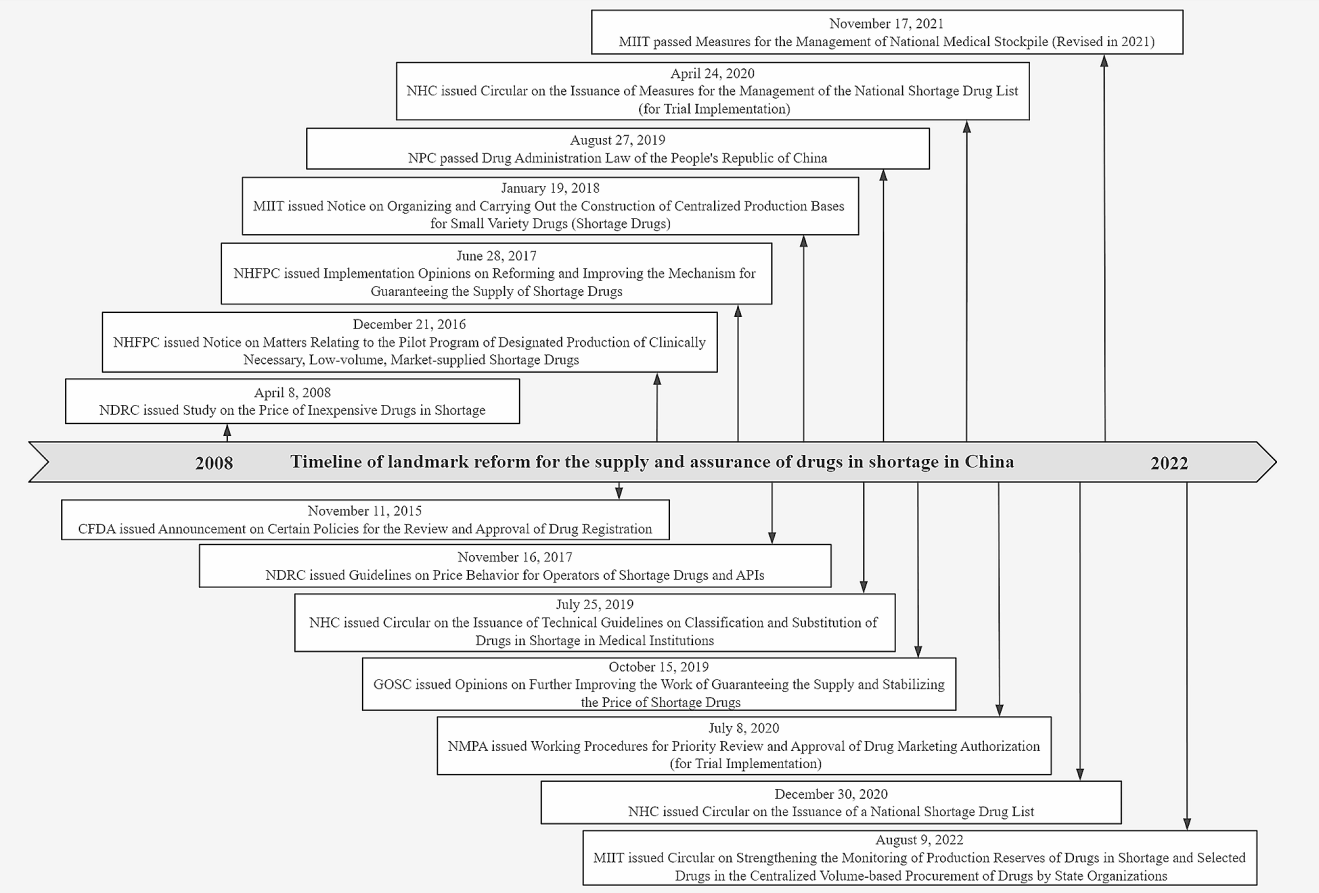
Timeline of landmark reform for the supply and assurance of drugs in shortage in China. (The publishing department is mainly the first responsible department: NDRC, National Development and Reform Commission; CFDA, China Food and Drug Administration (changed to the National Medical Products Administration in 2018); NHFPC, National Health and Family Planning Commission (changed to the National Health Commission in 2018); MIIT, Ministry of Industry and Information Technology; NHC, National Health Commission; NPC, National People’s Congress; GOSC, General Office of the State Council; NMPA, National Medical Products Administration)

Fortunately, China has initiated a series of reforms aimed at improving the supply security of medicines, as shown in Fig. 1. It can be observed that before 2015, China seldom introduced policies related to the supply and assurance of drugs in shortage, whereas, after 2015, China began to frequently carry out reforms to secure the supply and stabilize the price of the shortage of drugs. In particular, in the revised Drug Administration Law of the People’s Republic of China in 2019, a special chapter was made on drug stockpiling and supply [23]. This was the first time that China raised the regulation of the production of shortage drugs and the strengthening of drug supply guarantee to the legal level. In April 2020, the NHC clarified the definition of shortage drugs in China for the first time in the Measures for the Management of the National Shortage Drug List [13].In December 2020, the NHC released the first national-level list of shortage drugs and the key monitoring list of clinically necessary shortage-prone drugs [24]. The former recorded six drugs in severe shortage, namely Mitoxantrone, Methotrexate, Neostigmine, Benzylpenicillin, Sodium Thiosulfate, and Posterior Pituitary Injection; while the latter included 57 drugs, including 48 injections, 7 tablets, 1 capsule, and 1 oral solution. This key monitoring list of drugs in shortage has become an important basis for the subsequent work of ensuring the supply of medicines, such as the implementation of priority review and approval of the marketing authorization for drugs included in the list, and the discontinuation of the production reporting system. Shortage of drugs is a dynamic process of identification. In July 2022, four departments, including the Ministry of Industry and Information Technology (MIIT), jointly released the Shortage Drug Monitoring Varieties and Manufacturing Enterprises [25]. This is the second shortage drug monitoring list released in China, and it is also the most complete information and nationwide shortage drug monitoring list so far. This list records 980 drug preparations and 256 active pharmaceutical ingredients (APIs). Unlike the previous version of the list, this list directly lists the information of drug manufacturers and drug marketing authorization holders, which will help the regulator monitor the situation. More information on China’s policy documents on supply security of shortage drugs is shown in Table S1 of the Online Supplementary Document.

Despite previous studies on the current status of drug shortages and coping strategies in China. For example, Li analyzed the shortage of National Immunization Program vaccines in the city of Guangzhou, China, from 2017 to 2018, and found that insufficient production capacity was the primary reason [26]. Shukar found that sterile injectable products had a higher risk of shortages than other dosage forms of medicines for all medicines, and pointed out that medication shortages mainly consist of supply issues, demand issues, and regulatory issues [27]. Shi et al. developed a standardized decision-making process for drug shortage management and a therapeutic alternatives selection scheme, and then used the Delphi method and hierarchical analysis to establish an index system for evaluating the risk of drug shortages in healthcare institutions and empirically assessed the data on drug shortages in Hunan province [14, 28]. Song et al. analyzed the data of the shortage drug list of 24 provinces and cities based on the first shortage drug monitoring list released by China in 2020 [19]. However, it has to be admitted that almost all of the above studies have analyzed the current situation of drug shortages in some regions or individual provinces of China, and so far lack a description of the current situation of drug shortages in China as a whole. In addition, the drug shortage monitoring list issued by the Chinese government, as an important reference for drug supply assurance, is released only after comprehensively considering the drug shortage data reported by all provinces in the country, and it deserves to receive focused attention. Therefore, a study is urgently needed to reveal the full picture of the key drug shortage monitoring species in China.

In this study, we chose the Shortage Drug Monitoring Varieties and Manufacturing Enterprises released in July 2022 as the research data [25]. This is the most complete information and the most extensive coverage of the monitoring list of drugs in shortage released by Chinese officials so far. Through information extraction and entry to improve the information related to the monitored varieties, and then using descriptive statistical analysis, it aims to provide a comprehensive grasp of the current situation of drug shortages throughout China. This is the first study on monitoring varieties of drugs in shortage in China, which is of great significance in guiding the supply guarantee of drugs in shortage: (i) it helps to understand the characteristics of the attributes of drugs in shortage in China at present and the status of medication use for related diseases; (ii) it helps to find out the loopholes that exist in the listing approval of drugs in shortage and the production and supply capacity of the drugs; (iii) it helps to put forward targeted suggestions for improving the supply guarantee of drugs.

## Methods

### Data sources and extraction

This is a cross-sectional study on drug shortage monitoring varieties in China. The data on drug varieties were obtained from the list of Drug Shortage Monitoring Varieties and Manufacturing Enterprises, which was jointly released by four departments including the MIIT in July 2022 [25]. This list was released by the Chinese government after taking into account the shortage monitoring data reported by provinces and municipalities, and it is the most recent and most comprehensive shortage monitoring list released by the Chinese government so far, reflecting the current situation of drug shortages in China. This list consists of two tables, one is a total of 980 drugs from different manufacturers, and the other is a total of 256 APIs from different manufacturers. Then, the public data of the National Medical Products Administration (NMPA, https://www.nmpa.gov.cn/) and the Yaozhi Database (https://db.yaozh.com/) were queried to obtain the detailed information of the above varieties.

When refining the information on 980 drug preparations, five main aspects of the preparation’s characteristics were considered, namely, the drug manufacturer and the region to which it belongs, the classification of the drug type and dosage form, the classification of the drug anatomical therapeutic chemical (ATC) and clinical use, the status of the medical catalog to which the drug belongs, and the status of the number of drug approvals and production capacity. Drug manufacturers and the regions they belong to mainly examine the situation of domestic production and import of drugs, as well as the distribution of production enterprises in China. The classification of drug types and dosage forms examines which types of drugs (chemicals, traditional Chinese medicines, and biological products) are in great shortage, and which dosage forms are prone to shortage. Drug ATC and clinical use classification is based on the assessment of drug ATC codes and clinical use to understand the current stage of clinical drug application and shortage situation. The medical catalog to which the drugs belonged documented whether the drugs were listed in the National Drug Catalog for Basic Medical Insurance, Workers’ Compensation Insurance, and Maternity Insurance (hereinafter referred to as the National Medical Insurance Catalog) released in 2023 and the National Essential Drug Catalog in 2018 [29, 30]; whether they were medicines for children and rare diseases [31, 32]; whether they were included in the Volume-based procurement of drugs; and whether they were included in the first edition of the National Key Monitoring List of Clinically Necessary and Susceptible to Shortage Drugs in 2020. This is because the drugs included in the above lists are those that are of special concern to the regulatory authorities or have high requirements for drug accessibility. We also assessed the number of drug approvals and production capacity to understand the production and supply capacity of listed drugs in China. When populating the information of 256 APIs, we mainly considered the production and supply capacity of APIs, including the number of manufacturers of the same product in China, the status of co-review and approval with preparations, and whether they were included in the monitoring list of 57 key varieties to be released in 2020 [24]. All of the above information is included from the perspective of shortage drug regulation, from the characteristics of drug attributes, clinical use feedback, production, and supply capacity, and then assessed, which is conducive to subsequent targeted analysis and suggestions.

The data extraction process was initiated in January 2024 and was completed independently by two researchers and cross-checked with each other, with any disagreements encountered being discussed and agreed upon by all researchers in a consensus meeting of the research team.

### Data analysis

After nearly two months, the data of 980 drugs and 256 APIs included in the list were statistically analyzed after information refinement and checking for errors. This study used descriptive statistical analysis, using EXCEL2022 to analyze the research data for frequency (percentage) and other counting information, and if necessary, using Origin 2019b for graphing, to conclude the current situation of drug shortages in China, the attributes and characteristics of shortage medicines, and the ability to guarantee the supply of shortage drugs.

### Patient and public involvement

All material in this study was derived from official public data and open-access information and therefore did not require a research license or ethics committee assessment. Appropriate standards of scientific practice and research ethics were followed throughout the study.

## Results

### Analysis of the regions where the enterprises belonging to pharmaceutical products are located

Among the 980 drugs included in this list, 968 (98.78%) were produced in mainland China, while the remaining 12 (1.22%) drugs were imported. The manufacturers or marketing authorization holders (MAH) to which these medicines belong are distributed in 29 provinces, municipalities, and autonomous regions, except for the Tibet and Ningxia Autonomous Regions. Among them, Jiangsu (102, 20.41%), Henan (83, 8.47%), Shandong (63, 6.43%), Shanghai (61, 6.22%), and Guangdong (59, 6.02%) ranked in the top five. For the 256 APIs included in the priority monitoring list, all of them were produced in mainland China. These API manufacturers are mainly located in 26 provinces, municipalities, and autonomous regions except Guizhou, Nei Mongol, Tibet, Ningxia, and Xinjiang. Among them, Jiangsu (32, 12.50%), Beijing (26, 10.16%), Zhejiang (25, 9.77%), Shanghai (22, 8.60%), and Hubei (16, 6.25%) are among the top five. It can be seen that both APIs and drug preparations included in the monitoring are mainly concentrated in North China, the eastern coastal areas, and the southern cities, which coincides with the regional distribution of pharmaceutical enterprises in China.

### Classification of drug types and dosage forms

Among the 980 drug preparations, excluding four biological products from different enterprises (human prothrombinogen complex (2), recombinant human urokinaseogen for injection (1), and lyophilized human thrombin for external use (1)), all of them were chemical drugs, and no varieties of traditional Chinese medicine were included in the monitoring. In terms of drug dosage forms, a total of five dosage forms were involved: injections, tablets, capsules, oral solutions, and dispersions, the specific quantities of which are shown in Table 1. It can be seen that injections dominated the monitoring of these drugs.

**Table 1:** Classification of dosage forms of the 980 drugs in shortage included in the monitoring list

Drug dosage form	Numbers	Percentage
Injection	908	92.65%
Tablet	47	4.80%
Capsules	1	0.10%
Oral solution	2	0.20%
Powder medicine	22	2.24%

### ATC classification and clinical use classification of drugs

**Fig. 2:**
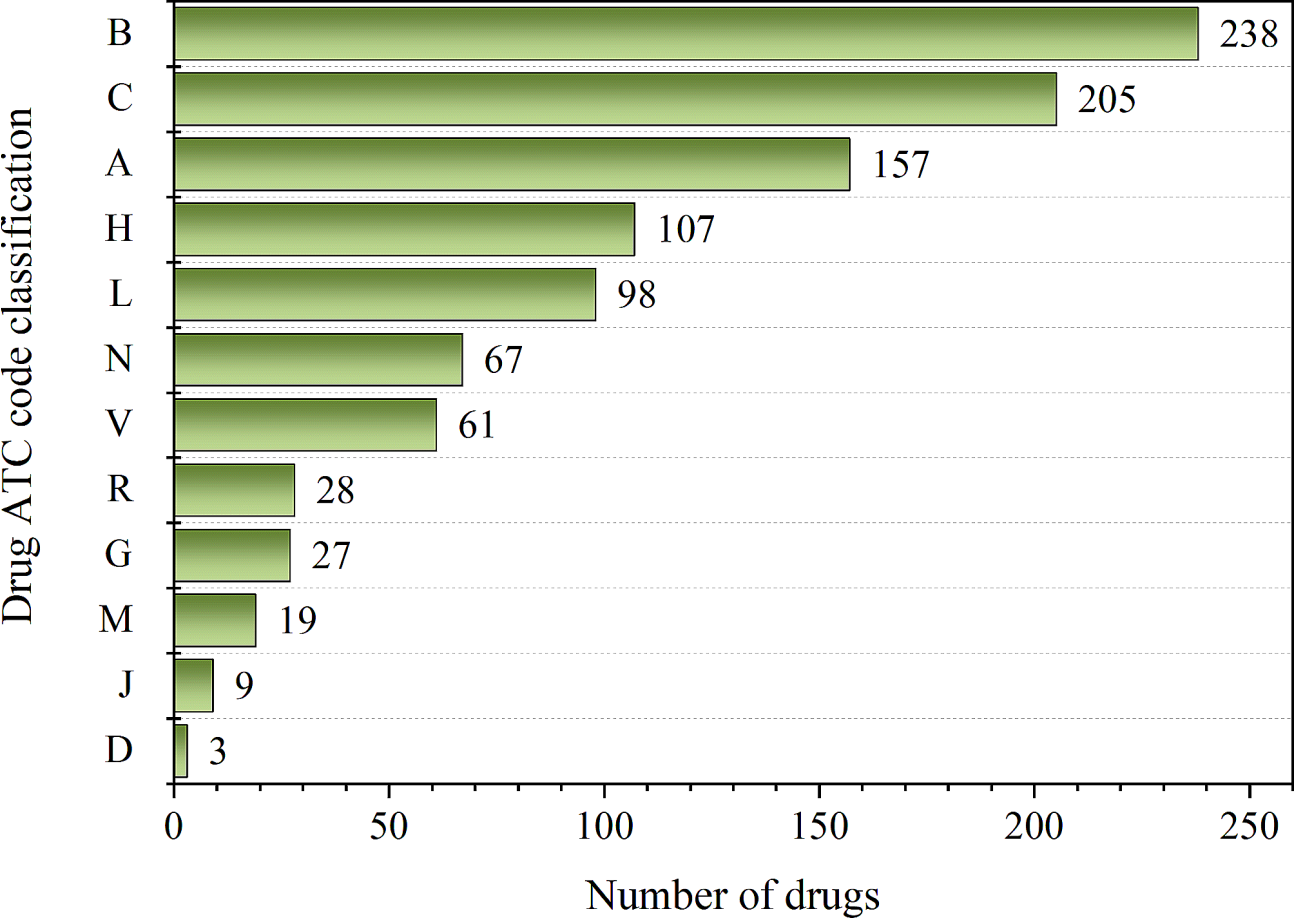
ATC classification of drugs in shortage that were included in monitoring (**A**): Alimentary tract and metabolism; (**B**): Blood and blood forming organs; (**C**): Cardiovascular system; (**D**): Dermatological; (**G**): Genito urinary system and sex hormones; (**H**): Systemic hormonal preparations, excluding sex hormones and insulins; (**J**): Antiinfective for systemic use; (**L**): Antineoplastic and immunomodulating agents; (**M**): Musculoskeletal system; (**N**): Nervous system; (**R**): Respiratory system; (**S**): Sensory organs; (**V**): Various

According to the Anatomical Therapeutic and Chemical (ATC) classification system set by WHO, the ATC classification of drugs in shortage is ordered as shown in Fig. 2. It can be seen that the top three in order are: drugs for blood and hematopoietic organs (238, 24.29%), drugs for the cardiovascular system (205, 20.92%), and drugs for digestive tract and metabolism (157, 16.02%). and drugs for the digestive tract and metabolism (157, 16.02%). It is important to note that four medicines (Compound Mannitol Injection, Magnesium Sulfate Injection, Tretinoin Tablets, and Magnesium Sulfate for Injection) are listed in different ATC codes because they can be used for the treatment of different categories of diseases (Table S2). For example, Compound Mannitol Injection is classified as B (Blood and Hematopoietic Organ Drugs) when it is used for cerebral edema and symptoms of high intraocular pressure, and A (Alimentary Tract and Metabolism Drugs) when it is used for cirrhotic ascites. Similarly, there are multiple classification codes for Tretinoin Tablets and Magnesium Sulfate Injection.

In addition, according to the clinical use of drugs, the NMPA had set up a specialized secondary catalog classification of drugs based on ATC codes, which was commonly used to select national essential drugs and drugs reimbursed by medical insurance. According to this classification, the 980 drug shortage monitoring varieties are mainly used in major diseases, first-aid or life-saving, public health, and other areas. Among them, the most drugs were used in the nervous system (211, 21.53%); the second most drugs were used in the cardiovascular system (143, 14.59%); and the fewest drugs were used in dermatology, with only three, and all of them were Tretinoin Tablets. The detailed classification of the clinical use of drugs is shown in Table 2, and some drugs have more than one clinical use and are therefore included in different catalogs.

**Table 2:** Classification of clinical uses of the 980 drugs in shortage included in the monitoring list

First class	Second class	Numbers	Percentage
Nervous system drugs	Drugs for cerebrovascular disease and lowering of cranial pressure	158	16.12%
	Central nervous system stimulants	38	3.88%
	Anti-myasthenia gravis drugs	9	0.92%
	Antiepileptic drugs	6	0.61%
Cardiovascular system drugs	Antishock drugs	72	7.35%
	Antihypertensive drugs	45	4.59%
	Antiarrhythmic drugs	21	2.14%
	Anti-heart failure drugs	5	0.51%
Digestive system drugs	Gastrointestinal antispasmodics and gastric stimulants	90	9.18%
	Diuretics and dehydrating drugs	20	2.04%
	Adjuvant therapy drugs for liver disease	28	2.86%
Antineoplastic drugs	Anti-metabolite drugs	32	3.27%
	Alkylating agents	23	2.35%
	Antitumor antibiotics	19	1.94%
	Other antitumor drugs	16	1.63%
	Antitumor botanical ingredients	9	0.92%
Hormones and endocrine-affecting drugs	Adrenocorticotropic hormone analogs	55	5.61%
	Thyroid hormones and antithyroid drugs	16	1.63%
Blood system drugs	Procoagulant	40	4.08%
	Anticoagulants and thrombolytics	30	3.06%
Antidotes	Antidotes for opioid poisoning	38	3.88%
	Antidotes for organophosphate poisoning	8	0.82%
	Antidotes for cyanide poisoning	4	0.41%
	Antidotes for nitrite poisoning	4	0.41%
	Antidotes for heavy metal poisoning	3	0.31%
	Antidotes to rat poison	1	0.10%
Vitamin and mineral drugs	Minerals	52	5.31%
Urologic system drugs	Diuretics and dehydration drugs	42	4.29%
Obstetrics and gynecology drugs	uterotonics	40	4.08%
Analgesic, antipyretic, anti-inflammatory, anti-rheumatic, anti-gout drugs	Anti-gout drugs	16	1.63%
	Antipyretic, analgesic, anti-inflammatory, anti-rheumatic drugs	10	1.02%
	Analgesic	1	0.10%
Drugs for mental disorders	Anxiolytic	20	2.04%
Anesthetic	Local anesthetic	14	1.43%
Anti-microbial drugs	Penicillins	6	0.61%
	Quinolones	2	0.20%
	Antileprosy	1	0.10%
Dermatologic drugs	Other dermatologic drugs	3	0.31%

### Incorporation into various medical catalogs

WHO published the first Essential Medicines Catalog in 1977, and China published the first National Essential Medicines Catalog in 2009 to actively respond to the WHO’s call for developing the essential medicines catalog, which is currently being implemented in the 2018 revised version [30]. Medicines included in the National Essential Medicines List are usually recognized as medicines that are adapted to basic healthcare needs, have appropriate dosage forms, are reasonably priced, can guarantee supply, and are equitably accessible to the public [33, 34]. However, the data reflected that of the 980 drug preparations included in the monitoring, 886 drugs were essential drugs, accounting for 90.41%.

In addition, to protect the basic medical needs of insured persons, China formulated the basic medical insurance drug catalog, which includes drugs that meet the basic conditions of clinical necessity, safety and effectiveness, and reasonable price into the medical insurance drug catalog [35]. The current implementation is the 2023 version of the catalog [29]. Medical insurance drugs can be categorized into Part A and Part B. Drugs in Part A are those that are clinically necessary for treatment, widely used, have proven efficacy, and are less expensive to treat than other drugs in their category. When a patient uses these medicines, all of them are included in the scope of reimbursement, after which they are reimbursed by the prescribed proportion. Drugs in Part B refer to drugs that are available for clinical treatment and are slightly more expensive than drugs in Part A in the same category. When patients use these drugs, they need to pay a certain percentage of the cost out of their own pockets, and the rest of the cost will be reimbursed to them according to the prescribed percentage [36, 37]. As can be seen from Table 3, among all the drugs included in the monitoring of shortage drugs, there were 881 drugs belonging to Part A, accounting for 89.90%; 51 drugs belonging to Part B, accounting for 5.20%; and 48 drugs not included in medical insurance, accounting for 4.90%.

**Table 3:** 980 drugs in shortage included in the monitoring list belonging to various pharmaceutical catalogs

List of medicines	Yes		No
National Essential Drug List	886 (90.41%)		61 (6.22%)
National Medical Insurance Drug List	Part A: 881 (89.90%)	Part B: 51 (5.20%)	48 (4.90%)
National Key Monitoring List of Clinically Essential Drugs Vulnerable to Shortage	980 (100%)		0 (0)
Volume-based procurement of drugs	25 (2.55%)		955 (97.45%)
Drugs for rare diseases	144 (14.70%)		836 (85.31%)
Drugs for children	412 (42.04%)		568 (57.96%)

Since 2019, China has started the work of volume-based procurement of drugs, adopting the method of volume-price linkage and volume-for-price exchange, and negotiating with drug manufacturers to achieve the reduction of the inflated prices of drugs and reduce the burden of medicine on patients under the premise of strictly guaranteeing the quality [38]. As of February 2024, the volume-based procurement has been implemented in the 9th batch. Among the monitored shortage drugs, 25 drugs from different enterprises were included in the volume-based procurement list, which contained eight kinds of drugs in fact: Furosemide injection, Carbetocin injection, Oxytocin Injection, Atropine Sulfate injection, Calcium Gluconate injection, Amiodarone Hydrochloride injection, Magnesium Sulfate injection, and Mannitol injection.

In 2020, the NHC released the National Key Monitoring List of Clinically Essential Drugs Vulnerable to Shortage, which contained a total of 57 drugs [24]. Unlike the key monitoring varieties released at this time, the 57 drugs in the 2020 version only had the drug names and did not indicate the manufacturing companies that needed to be monitored. Therefore, after checking the 980 drugs in this edition with the 57 drugs released previously, we found that the list of 980 drugs being monitored this time was derived from the 2020 edition. Except for nitroglycerin and antivenom, all preparations corresponding to the 57 drugs in the 2020 version were recorded in the 2022 version of the shortage monitoring list. As of the time of data entry, nitroglycerin had 49 drug approvals in China, corresponding to 33 drug manufacturers, and the production volume of the drug was able to meet the demand for the drug, and it was discharged from the shortage drug monitoring list. Antivenom has not been produced by any enterprise since 2022 and is currently in a state of discontinued production. A comparison of the drug information between the 2020 version of the monitoring list of clinically necessary drugs in shortage and the monitoring list of shortage drugs in 2022 is shown in Table S3.

Furthermore, we have also paid attention to the demand for medicines for special populations. Medicines for rare diseases and children have been emphasized by the Chinese government in recent years, and it has been proposed to strengthen the supply of medicines for rare diseases and for children in a dozen governmental work reports. In terms of rare diseases, China currently does not have an official definition and identification criteria for rare diseases or orphan drugs and only uses the two batches of rare disease catalogs released by the NHC as the basis for identification [31, 32]. We matched the rare disease catalogs with the shortage drug monitoring list and found that 144 (14.7%) of the monitored shortage drugs were used to treat rare diseases. In terms of medicines for children, we checked the drug instructions of 980 drug preparations one by one and found that 412 (42.04%) medicines had clear instructions for children.

### Analysis of the number of drug approvals and production capacity

As shown in Table 4, among the 980 monitored drugs in shortage, the number of approvals is less than 10 in total 209 drugs, accounting for 21.33%. Among them, there were 20 drugs with only one number of approvals in China, as shown in Table S4 (No.1–20). In terms of the number of manufacturers, there were 232 drugs with less than 10 manufacturers, accounting for 23.67%; while there were 143 drugs with less than 5 manufacturers, accounting for 14.59%. It is noteworthy that 26 drugs are produced by only one enterprise in China, as shown in Table S4 (No.1–26). In addition, Table S3 also showed that as of the time of data entry, the number of manufacturers of seven products, including Digoxin Oral Solution, Furosemide for Injection, and Methylene Blue Injection, had decreased compared to the time of the release of the list, and there were cases of discontinuation and delisting. Moreover, there were some drugs with a huge gap between the number of manufacturers and the number of approvals, such as Thrombin Lyophilized Powder, Urokinase for Injection, Atropine Sulfate Injection, Mannitol Injection, Hydrocortisone Injection, etc. This was because some of the producers withdrew from the market due to the fierce market competition and lower profits after obtaining the drug approvals, which led to a significant reduction of actual producers [39].

**Table 4:** Production and supply capacity of drugs and APIs included in the monitoring

Number of producers	Drug preparations	APIs
≥10	748 (76.33%)	104 (40.63%)
≥5-<10	89 (9.08%)	80 (31.25%)
≥2-<5	117 (11.94%)	67 (26.17%)
Only 1	26 (2.65%)	5 (1.95%)

### Analysis of the supply capacity of APIs

First of all, we combed through the sources of APIs of the 980 drugs included in the monitoring and found that the manufacturing enterprises of 873 drugs needed to purchase the APIs of those drugs externally, which accounted for 89.08%, while only 107 (10.92%) of the enterprises of the drugs could produce the APIs by themselves.

Additionally, the NHC in announcing this shortage drug monitoring list, also announced for the first time the monitoring varieties of APIs, covering a total of 256 APIs from different manufacturers, which were 61 types of APIs. After combing through this list of APIs, we found that 152 APIs have less than 10 manufacturers in China; 72 APIs have less than 5 producers and even 5 kinds of APIs have only one production in China, which are: Dibromomannan, Corticotropin, Sodium Dimercaptopropanesulfonate, Bleomycin Hydrochloride and Mitoxantrone, and the specific information is shown in Table 4 and Table S5.

## Discussion

This paper is the first cross-sectional study of the current situation of drug shortages in China based on the shortage monitoring data released at the national level in China. Through the study, we understood the current status of drug shortages in China and gained a clear view of the attributes and main characteristics of the drugs included in the drug shortage monitoring list.

Similar to the types of drugs in shortage in countries such as the United States, Poland, and France [40, 41], the number of chemical drugs and injections accounted for the largest proportion of shortage drugs in China. According to the study statistics, 976 of the 980 shortage drugs included in the monitoring list were chemical drugs, accounting for 99.59%. This is mainly related to the fact that chemical drugs are easy to stockpile, convenient to take, and have high market demand and clinical use. Similar to the study of Shukar et al. we found that drug dosage forms were mainly concentrated in injections, and there were 908 injections with shortages, which accounted for 92.7% of the number [24]. Studies have shown that the production process of injectables is complex and requires high equipment, and the production process is characterized by a long cycle time and high risk, which makes it more prone to shortages [42]. In addition, most of the shortage drugs are cheap drugs, resulting in insufficient profits for enterprises, in which case injectables are more costly than other dosage forms and are more likely to experience shortages [43].

In terms of drug therapeutic areas, there were major shortages of drugs for blood and hematopoietic organs, the cardiovascular system, gastrointestinal tract, and metabolism, as well as the neurological system. As it happens, most of these drugs are in turn considered by us as life-saving and clinically essential drugs [44, 45]. For example, Norepinephrine Bitartrate Injection and Epinephrine Hydrochloride Injection, both of which are cardiovascular system drugs commonly used clinically for severe respiratory distress due to bronchospasm, cardiac arrest, infiltrative anesthesia, anaphylaxis, and treatment of hypotension, have experienced shortages of varying degrees from 2016 to the present day in several regions of China. Moreover, we also found that there were also shortages of clinical emergency drugs in foreign countries. Lin et al. concluded in a cross-sectional study that there were shortages of clinical emergency drugs such as Ondansetron, Hydromorphone, and Promethazine in the United States [46]; Angelis et al. pointed out that there were shortages of clinical life-saving drugs such as Aspirin in the United Kingdom as well [47]. Emergency drugs are difficult to replace with other drugs in the clinic, and if there is a shortage, patients are likely to miss the best time for treatment as a result. Compared with other drugs, although these drugs are life-saving drugs and clinically necessary drugs, the clinical usage is small, and the price is low for a long time [27, 44]. Therefore, under the influence of market competition, many pharmaceutical companies have a low willingness to produce, and to survive, they have to give up some of the drugs with small profit margins and small production quantities, which leads to a gradual decrease in the supply of some emergency drugs, and the phenomenon of undersupply and unavailability of drugs [48]. Drugs such as this clinical necessity, small usage or low transaction price, insufficient enterprise production incentive, and other supply shortages, can be guaranteed through market matching, designated production, unified distribution, included in the stockpile, and other measures to ensure production and supply [49].

We further assessed the inclusion of the monitored varieties in the respective medicine catalogs. National essential medicines and medicines in the national medical insurance catalog are essential, safe, and effective medicines selected for prevention and treatment based on China’s basic national conditions at the current stage and the ability of the basic medical insurance system to protect them, and are characterized by reasonable prices, a large proportion of medicines, and mostly commonly used in clinical practice, so that, in principle, there should be no shortages [50]. However, data feedback showed that more than 90% of the medicines included in the monitoring were essential medicines, and more than 95% of the medicines in shortage were included in the national medical insurance catalog. The reasons for the shortage of national essential drugs and medical insurance drugs are, on the one hand, due to the low price and insufficient supply of drugs; on the other hand, it is attributed to the continuous emergence of new drugs, which have replaced some of the essential drugs, resulting in the reduction or even disappearance of their use. Therefore, the governmental departments need to further improve the level of supply guarantee for essential drugs and medical insurance drugs [41].

On the contrary, we find that a much smaller proportion of the monitored varieties of drugs in shortage were included in the drug catalog for volume-based procurement than in the other catalogs. This is because the drug volume-based procurement model enables cross-regional purchasing alliances and cross-provincial adjustments. Its biggest advantage is that it guarantees the number of transactions and forces enterprises to report their production capacity, inventory, supply, etc., which guarantees that the procurement needs of medical institutions for the selected drugs will be met promptly during the procurement cycle [51, 52].

In addition, in our study, we specifically focused on medicines for rare diseases and for children. The results showed that among the drugs included in the monitoring, drugs for rare diseases accounted for 14.70%, and drugs for children accounted for 42.04%. We have to admit that compared with developed countries in Europe and the United States, the current access to orphan drugs and medicines for children is inherently difficult in China, which is not only related to the cost of research and development of orphan drugs and medicines for children, but also related to the high risk of participation in clinical trials for children or patients with rare diseases, and the difficulty of recruiting subjects and other factors [53–55]. Therefore, the shortage of such a high percentage of drugs available for pediatric and rare disease populations further suggests that the government should implement the rules of supportive policies for the development of pediatric and orphan drugs to guarantee the supply of such drugs.

Finally, we focused on analyzing drug production and supply capacity, which has received little attention in other studies. Our findings revealed that the low number of drug producers and the low number of drug approvals were potential causes of drug shortages. The data fed back that 26 medicines are exclusively produced in China, and even 20 medicines have only one number of approvals in China. Some of these drugs were used for first aid or rescue medicines for organophosphorus pesticide poisoning, acute ST-segment elevation myocardial infarction, and postoperative bleeding. For this kind of drugs that are produced by only one enterprise or have insufficient production capacity, market supply, and demand and imbalance, on the one hand, the government must strengthen market supervision, improve the production and supply capacity of the relevant raw materials and auxiliary materials, increase the monitoring of production and supply of pharmaceutical enterprises, and introduce a multi-channel drug reserve supply mechanism [48]. On the other hand, the government must give certain policy support and technical support, implement the priority review and approval procedures for drugs in shortage, accelerate the production and approval qualifications of relevant drugs, and improve the production capacity of drugs [56].

The study also found that nearly 90% of enterprises needed to purchase the APIs necessary to produce preparations. At the same time, the low production capacity of APIs and the monopoly of APIs were relatively obvious. The shortage or monopolization of APIs has led to insufficient production capacity of manufacturers, resulting in the phenomenon of drug shortage has been pointed out in some studies [22, 26, 27]. China’s approval of API production is very strict, and the relevant laws stipulate that drug manufacturers can only use APIs with authorization [57]. In the case of APIs such as Dibromomannan, Corticotrophin, Sodium Dimercaptopropanesulfonate, Bleomycin Hydrochloride, Mitoxantrone, which were produced by only one manufacturer, once the manufacturer suspends or terminates the production for some special reasons, a shortage of the APIs will be caused, which would directly lead to the inability of the production of preparations to carry out the work normally, and thus lead to a shortage of medicines. Furthermore, there also exists a collusion of interests between API enterprises and preparation enterprises in the market, which inflates the price of APIs and monopolizes the market. For example, in 2023, China’s State Administration for Market Regulation issued a fine, publicizing that Yuanda Pharmaceutical (China) and Wuhan Huihai illegally carried out the monopoly of metanephrines APIs and epinephrine APIs, which resulted in a shortage of medicines and jeopardized patients’ rights and interests and the public interest of the society [58].

Summarizing the above discussion and taking into account the current public health needs of China, we put forward the following recommendations for strengthening the supply security of medicines in shortage in China:

(i) Accelerating the dynamic updating of the drug shortage monitoring list.

Unlike the FDA, which releases drug shortage data to the public every year, drug shortage data in China is usually released irregularly, and the latest release was two years ago. However, we cannot deny that the shortage drug monitoring list is an important reference for guiding the main bodies to participate in drug supply [59]. Therefore, we suggest that the system of dynamically updating the list of medicines in shortage should be improved and that the catalog should be optimized and updated promptly, based on changes in regional demographics and disease spectrums, to satisfy the clinical demand for drugs. Smooth the existing channels for monitoring information on drug shortages, promote the construction of a big data platform related to drug supply, and broaden other monitoring channels, such as industry supervision and public oversight, to further realize the informatization of drug shortage supervision.

(ii) Promoting stockpile management of emergency drugs and sole production drugs.

The 980 drugs and 256 APIs included in the list have different shortages. For emergency drugs and sole proprietary drugs in urgent clinical need, the integrated development of key APIs and preparations should be promoted, and the stockpiling of drugs should be accelerated to ensure a multi-channel stockpile supply [48, 49]. In the revised version of the Measures for the Management of National Pharmaceutical Stockpile in 2021, it is proposed to carry out the model of combining physical stockpiling and production capacity stockpiling for clinically necessary and shortage-prone medicines [60]. At the same time, a new model of government-enterprise synergistic shortage drug reserve can also be explored. Recently, the Shandong Provincial Government commissioned China Resources Pharmaceutical Group Limited to carry out the shortage of drug reserves, realizing the integration of production, reserve, and supply, and alleviating the pressure of government storage.

(iii) Improving the review and approval of shortage drugs.

The Drug Review Center (CDE) of the NMPA started to implement the Priority Review and Approval Work Procedure for Drug Marketing Authorization in 2016, whereby clinically vulnerable drugs in shortage can be included in the Priority Review and Approval Procedure (PRA) [61]. We also found that more than 200 clinically vulnerable shortage medicines have been accelerated to the market through PRA since the release of the list when we conducted an information search in the Yaozhi Database. Next, the requirements of the Drug Administration Law of the People’s Republic of China to guarantee the supply of drugs should be further implemented to improve the review and approval system for shortage drugs, to accelerate the listing of shortage drugs eligible for priority review as soon as possible to meet the market demand.

(iv) Implementing a record-keeping system for the discontinuation of drugs in shortage.

We have found that some drugs in shortage (e.g., antivenom) have even been out of stock on the market due to the scarcity of supplying enterprises. In this regard, it is recommended to strengthen the monitoring and early warning of the supply guarantee of shortage medicines nationwide, strictly implement the filing system for the discontinuation of shortage drugs and APIs, increase the strength of drug reserves, and make a good coping strategy beforehand, to ensure that medicines are not unavailable to patients.

Our study has some limitations. First, the ATC classification and clinical use classification of the listed drugs in this study mainly considered the main indications of the drugs. In the case of drug shortages, some drugs are also used off-label in the clinical setting, which is not considered in this paper. Secondly, drug shortages are also related to factors such as enterprise production costs and drug prices, but these data cannot be fully obtained through public data, so it is not possible to analyze in-depth the situation of insufficient willingness to produce due to low profits. Thirdly, the current situation of drug shortages in different provinces is related not only to drug production but also to drug distribution. Because of the inability to obtain drug distribution information, it is not possible to analyze the differences in drug shortages in different regions. Fourth, China does not regularly release data on drug shortages, and the data analysis in this paper represents only one point in time, so our study cannot fully analyze the trend of drug shortages and the effectiveness of related policies. Our team will continue to promote research on the supply security of drug shortages, and we hope to remedy the above limitations in the future.

## Conclusion

In this paper, based on the 980 drug preparations and 256 APIs in the Shortage Drug Monitoring Varieties and Manufacturing Enterprises released by the MIIT 2022, we used public databases to obtain detailed information about the drugs, and then analyzed and discussed China’s drug shortage profile through descriptive statistical analysis. The shortage of injectables was very obvious in China’s shortage of drugs, and there were large shortages of drugs for blood and hematopoietic organs, cardiovascular system, digestive tract and metabolism, and neurological system, as well as a large number of shortages of essential medicines, medical insurance medicines, medicines for children, and medicines for rare diseases, and some of the production capacity of some drugs and the supply capacity of APIs need to be improved. We recommend that government authorities and pharmaceutical companies work together to strengthen the communication of information on drug shortages, do a good job of monitoring and early warning of drug shortages, and strengthen the management of purchasing and stockpiling processes to ensure that drugs are equitably accessible and that patients’ health rights and interests are safeguarded. This study informs all industry insiders about the current situation of drug shortages in China and provides a reference for improving the drug shortage situation.

### Electronic supplementary material

Below is the link to the electronic supplementary material.

Supplementary Material 1

## Data Availability

Data is provided within the manuscript or supplementary information files.

## Abbreviations

API: Active pharmaceutical ingredient
ATC: Anatomical Therapeutic Chemical
CDE: Drug Review Center
CFDA: China Food and Drug Administration
EU: European Union
FDA: Food and Drug Administration
GOSC: General Office of the State Council
MIIT: Ministry of Industry and Information Technology
NDRC: National Development and Reform Commission
NHC: National Health Commission
NHFPC: National Health and Family Planning Commission
NMPA: National Medical Products Administration
NPC: National People’s Congress
PRA: Priority Review and Approval
UK: United Kingdom
WHO: World Health Organization
