# Supplementary material for: Drug supply and assurance: a cross-sectional study of drug shortage monitoring varieties in China
Source: BMC Public Health. 2024 Jul 30;24:2048. doi: 10.1186/s12889-024-19361-5 (PMC11289944; doi:10.1186/s12889-024-19361-5)
Supplement: Supplementary file 1 — Supplementary Material 1 [file 12889_2024_19361_MOESM1_ESM.pdf]

## ONLINE SUPPLEMENTARY DOCUMENT

**Title:** Drug supply and assurance: a cross-sectional study of drug shortage monitoring varieties in China


**Authors:** Yipeng Lan[1], Xiaofeng Lin[1], Qiannan Chen[1], Li Wang[1], Lihua Sun[1,2*], Zhe Huang[1,2*]
1 School of Business Administration, Shenyang Pharmaceutical University, Shenyang, China
2 Institute of Drug Regulatory Science, Shenyang Pharmaceutical University, Shenyang, China
* Correspondence: Zhe Huang (huangzhe2000@sina.com); Lihua Sun (slh-3632@163.com)


**Table S1** Reform of China's Shortage Drug Supply Security Policies

| No. | Release time | Policy title | Publishing department | Key content on security of supply of shortage drugs |
|---|---|---|---|---|
| 1 | April 8, 2008 | Study on the Price of Inexpensive Drugs in Shortage | NDRC | The situation of the shortage of inexpensive drugs and the reasons for the shortage of drugs in China from 2002 to 2007 are explained. |
| 2 | November 11, 2015 | Announcement on Certain Policies for the Review and Approval of Drug Registration | CFDA | Encourage the research development and production of shortage drugs in the market, establish a regular communication mechanism for shortage drugs, and put forward proposals to speed up the approval of shortage drugs. |
| 3 | December 21, 2016 | Notice on Matters Relating to the Pilot Program of Designated Production of Clinically Necessary, Low-volume, Market-supplied Shortage Drugs | NHFPC | Proposing to carry out pilot projects for the designated production of clinically necessary medicines, in small quantities and short supply in the market, and completing the bidding process for the designated production of three varieties of Digoxin Oral Solution, Compound Sulphamethoxazole Injection, and Sodium Para-Aminosalicylate for Injection. |
| 4 | June 28, 2017 | Implementation Opinions on Reforming and Improving the Mechanism for | NHFPC | Measures such as improving the system of monitoring and early warning of drug shortages and list management, establishing a |

| | | | | |
|---|---|---|---|---|
| | | Guaranteeing the Supply of Shortage Drugs | | hierarchical linkage response mechanism for the supply and security of drugs in shortage, and implementing a categorized and precise policy for the supply and security of shortage drugs have been stipulated. |
| 5 | November 16, 2017 | Guidelines on Price Behavior for Operators of Shortage Drugs and APIs | NDRC | It proposed measures to regulate the market price behavior of shortage drugs and APIs, maintain the market price order, and establish a fair and competitive market environment for the production, purchase, and sale of drugs and APIs. |
| 6 | January 19, 2018 | Notice on Organizing and Carrying Out the Construction of Centralized Production Bases for Small Variety Drugs (Shortage Drugs) | MIIT | Combined with the demand for drug supply security and the national layout of centralized production bases, about five enterprises will be selected and recognized for the construction of centralized production bases for small-variety drugs ( shortage drugs). |
| 7 | July 25, 2019 | Circular on the Issuance of Technical Guidelines on Classification and Substitution of Drugs in Shortage in Medical Institutions | NHC | Guiding medical institutions to carry out categorized and graded assessments of clinically necessary drugs in shortage, and carrying out the scientific selection of alternative medicines, to safeguard clinical therapeutic needs and standardize their use. It has standardized the assessment of information on the shortage of medicines and the selection of alternative medicines. |
| 8 | August 27, 2019 | Drug Administration Law of the People's Republic of China | NPC | A special chapter was devoted to the stockpiling and management of medicines, requiring the State to implement a list management system for shortage drugs. |
| 9 | October 15, 2019 | Opinions on Further Improving the Work of Guaranteeing the Supply and Stabilizing the Price of Shortage Drugs | GOSC | It is required to improve the sensitivity and timeliness of monitoring and response, to strengthen the provision and use of basic medicines and the standardized management of medication in medical institutions, and to improve the procurement of shortage medicines. |
| 10 | April 24, 2020 | Circular on the Issuance of Measures for the Management of the National Shortage | NHC | Proposed a management method for the national list of shortage drugs, issued a provincial (municipal, district, and county) information report |

| | | | | |
|---|---|---|---|---|
| | | Drug List (for Trial Implementation) | | on shortage drugs, and for the first time provided an official definition of shortage drugs. |
| 11 | July 8, 2020 | Working Procedures for Priority Review and Approval of Drug Marketing Authorization (for Trial Implementation) | NMPA | It has clarified that when applying for marketing authorization for medicines, shortage drugs that are urgently needed clinically will be included in the priority review and approval procedure. |
| 12 | December 30, 2020 | Circular on the Issuance of a National Shortage Drug List | NHC | Released China's first national list of shortage drugs (including 6 drugs) and the first national key monitoring list of clinically necessary drugs susceptible to shortage (including 57 drugs). |
| 13 | November 17, 2021 | Measures for the Management of National Medical Stockpile (Revised in 2021) | MIIT | Require a drug stockpiling model that combines various forms of stockpiling, such as physical stockpiling and production capacity stockpiling, for shortage drugs. |
| 14 | August 9, 2022 | Circular on Strengthening the Monitoring of Production Reserves of Drugs in Shortage and Selected Drugs in the Centralized Volume-based Procurement of Drugs by State Organizations | MIIT | Released China's second list of key monitoring varieties of shortage drugs, covering 980 drug preparations and 256 APIs, the most comprehensive list of key monitoring varieties of shortage drugs to date. |

Note: The publishing department here mainly refers to the department at the top of the list. NDRC, National Development and Reform Commission; CFDA, China Food and Drug Administration (changed to the National Medical Products Administration in 2018); NHFPC, National Health and Family Planning Commission (changed to the National Health Commission in 2018); MIIT, Ministry of Industry and Information Technology; NHC, National Health Commission; NPC, National People's Congress; GOSC, General Office of the State Council; NMPA, National Medical Products Administration

**Table S2 Detailed information on monitored drugs with multiple ATC codes**

| No. | Drug name | Number of enterprises included in monitoring | Indications | ATC classification |
|---|---|---|---|---|
| 1 | Compound Mannitol Injection | 14 | Cerebral edema, high intraocular pressure, hypoproteinemia | B (Blood and blood forming organs) |
| | | | Nephrotic syndrome, cirrhotic ascites | A (Alimentary tract and metabolism) |
| 2 | Magnesium Sulfate Injection | 21 | Hypertension in pregnancy, convulsions | C (Cardiovascular system) |
| | | | Pre-eclampsia, Eclampsia | G (Genito urinary system and sex hormones) |
| 3 | Tretinoin Tablets | 3 | Keratosis pilaris, lichen planus, multiple common warts, erythematous furuncle, acne | D (Dermatological) |
| | | | Oral mucosal leukoplakia, acute promyelocytic leukemia, ichthyosis, psoriasis | L (Antineoplastic and immunomodulating agents) |
| 4 | Magnesium Sulfate for Injection | 1 | Hypertension in pregnancy, convulsions | C (Cardiovascular system) |
| | | | Pre-eclampsia, eclampsia | G (Genito urinary system and sex hormones) |

**Table S3** Comparison of the 2020 version of the monitoring list and the 2022 version of the monitoring list, and the number of existing drug manufacturers

| No. | 2020 version of the National Key Monitoring List of Clinically Essential Drugs Vulnerable to Shortage | | 2022 version of the List of Drugs in Shortage Monitoring Varieties | | | Number of drug manufacturers in 2024 (as of March 2024) | Increase or decrease in the number of manufacturers |
|---|---|---|---|---|---|---|---|
| | Name of drug species | Dosage form | Generic name | Number of corresponding manufacturers/market authorization holders | Whether within the 2020 version of the list | | |
| 1 | Benzathine Benzylpenicillin | Injection | Benzathine Benzylpenicillin for Injection | 6 | Yes | 6 | 0 |
| 2 | Clofazimine | Capsule | Clofazimine Soft Capsules | 1 | Yes | 1 | 0 |
| 3 | Allopurinol | Tablet | Allopurinol Tablets | 14 | Yes | 14 | 0 |
| | | | Compound Allopurinol Tablets | 1 | Yes | 1 | 0 |
| | | | Allopurinol Sustained-release Tablets | 1 | Yes | 1 | 0 |
| 4 | Neostigmine | Injection | Neostigmine Methylsulfate Injection | 5 | Yes | 7 | +2 |
| | | | Neostigmine Methylsulfate for Injection | 1 | Yes | 1 | 0 |
| 5 | Pyridostigmine Bromide | Tablet | Pyridostigmine Bromide Tablets | 3 | Yes | 3 | 0 |
| 6 | Phenobarbital | Injection | Sodium Phenobarbital | 4 | Yes | 5 | +1 |

| | | | | | | | |
|---|---|---|---|---|---|---|---|
| | | | Injection | | | | |
| | | | Phenobarbital Sodium for Injection | 2 | Yes | 2 | 0 |
| 7 | Mannitol | Injection | Mannitol Injection | 144 | Yes | 149 | +5 |
| | | | Compound Mannitol Injection | 14 | Yes | 15 | +1 |
| | | | Oxaliplatin and Mannitol Injection | 3 | Yes | 3 | 0 |
| | | | Paracetamol and Mannitol Injection | 2 | Yes | 3 | +1 |
| | | | Fleroxacin and Mannitol Injection | 1 | Yes | 1 | 0 |
| | | | Ofloxacin and Mannitol Injection | 1 | Yes | 1 | 0 |
| 8 | Nikethamide | Injection | Nikethamide Injection | 18 | Yes | 29 | +11 |
| 9 | Lobeline | Injection | Lobeline Hydrochloride Injection | 10 | Yes | 12 | +2 |
| 10 | Diazepam | Injection | Diazepam Injection | 20 | Yes | 23 | +3 |
| 11 | Nitroglycerine | Tablet, Injection | - | - | No | 33 | - |
| 12 | Propafenone | Injection | Propafenone Hydrochloride Injection | 6 | Yes | 6 | 0 |
| 13 | Amiodarone | Injection | Amiodarone Hydrochloride Injection | 7 | Yes | 9 | +2 |
| | | | Amiodarone Hydrochloride For Injection | 1 | Yes | 2 | +1 |

| | | | | | | | |
|---|---|---|---|---|---|---|---|
| 14 | Verapamil | Injection | Verapamil Hydrochloride Injection | 6 | Yes | 9 | +3 |
| | | | Verapamil Hydrochloride for Injection | 1 | Yes | 1 | 0 |
| 15 | Digoxin | Oral Solution | Digoxin Oral Solution | 3 | Yes | 2 | -1 |
| 16 | Deslanoside | Injection | Deslanoside Injection | 3 | Yes | 3 | 0 |
| 17 | Sodium Nitroprusside | Injection | Sodium Nitroprusside for Injection | 11 | Yes | 11 | 0 |
| | | | Sodium Nitroprusside Injection | 1 | Yes | 1 | 0 |
| 18 | Magnesium Sulfate | Injection | Magnesium Sulfate Injection | 21 | Yes | 30 | +9 |
| | | | Magnesium Sulfate for Injection | 1 | Yes | 1 | 0 |
| | | | Magnesium Sulfate Glucose Injection | 1 | Yes | 1 | 0 |
| 19 | Phentolamine | Injection | Phentolamine Mesylate Injection | 7 | Yes | 8 | +1 |
| | | | Phentolamine Mesylate for Injection | 3 | Yes | 3 | 0 |
| 20 | Epinephrine | Injection | Epinephrine Hydrochlaride Injection | 22 | Yes | 23 | +1 |
| | | | Procaine and Adrenaline Injection | 11 | Yes | 13 | +2 |
| | | | Phenylephrine Hydrochloride Injection | 2 | Yes | 4 | +2 |
| | | | Articaine Hydrochloride and | 2 | Yes | 2 | 0 |

| | | | | | | | |
|---|---|---|---|---|---|---|---|
| | | | Epinephrine Tartrate Injection | | | | |
| | | | Mepivacaine Hydrochloride and Adrenaline Injection | 1 | Yes | 1 | 0 |
| 21 | Norepinephrine | Injection | Noradrenaline Bitartrate Injection | 7 | Yes | 11 | +4 |
| 22 | Isoproterenol | Injection | Isoproterenol Hydrochloride Injection | 2 | Yes | 2 | 0 |
| | | | Isoprenaline Sulfate Injection | 1 | Yes | 1 | 0 |
| 23 | Dopamine | Injection | Dopamine Hydrochloride Injection | 8 | Yes | 25 | +17 |
| | | | Dopamine hydrochloride for Injection | 2 | Yes | 2 | 0 |
| 24 | Dobutamine | Injection | Dobutamine Hydrochloride Injection | 23 | Yes | 26 | +3 |
| | | | Dobutamine Hydrochloride and Glucose Injection | 3 | Yes | 3 | 0 |
| | | | Dobutamine Hydrochloride for Injection | 2 | Yes | 2 | 0 |
| 25 | Atropine | Injection | Atropine Sulfate Injection | 88 | Yes | 98 | +10 |
| | | | Atropine Sulfate and Promethazine Hydrochloride Injection | 2 | Yes | 2 | 0 |
| | | | Morphine and Atropine Sulfate Injection | 1 | Yes | 1 | 0 |
| 26 | Arginine | Injection | Arginine Hydrochloride | 8 | Yes | 9 | +1 |

| | | | | | | | |
|---|---|---|---|---|---|---|---|
| | | | Injection | | | | |
| | | | Arginine Acetylsalicylate for Injection | 5 | Yes | 5 | 0 |
| | | | Arginine Hydrochloride for Injection | 4 | Yes | 4 | 0 |
| | | | Arginine Glutamate Injection | 1 | Yes | 1 | 0 |
| | | | Arginine Hydrochloride and Glucose Injection | 1 | Yes | 2 | +1 |
| 27 | Furosemide | Injection | Furosemide Injection | 58 | Yes | 66 | +8 |
| | | | Furosemide for Injection | 4 | Yes | 3 | -1 |
| 28 | Thrombin | Lyophilizing Powder | Lyophilizing Thrombin Powder | 19 | Yes | 19 | 0 |
| | | | Human Prothrombin Complex | 2 | Yes | 2 | 0 |
| | | | Thrombin | 2 | Yes | 2 | 0 |
| | | | Human Thrombin for External use,Freeze-dried | 1 | Yes | 1 | 0 |
| 29 | Vitamin K1 | Injection | Vitamin K1 Injection | 13 | Yes | 13 | 0 |
| 30 | Protamine | Injection | Protamine Sulfate Injection | 3 | Yes | 3 | 0 |
| 31 | Urokinase | Injection | Urokinase for Injection | 3 | Yes | 29 | +26 |
| | | | Recombinant Human Urokinase for Injection | 1 | Yes | 1 | 0 |
| 32 | Hydrocortisone | Injection | Hydrocortisone Injection | 48 | Yes | 57 | +9 |
| | | | Hydrocortisone Sodium Succinate for Injection | 4 | Yes | 4 | 0 |
| | | | Hydrocortisone Acetate | 1 | Yes | 1 | 0 |

| | | | | | | | |
|---|---|---|---|---|---|---|---|
| | | | Injection | | | | |
| 33 | Corticotropin | Injection | Corticotropin for Injection | 2 | Yes | 2 | 0 |
| 34 | Thiamazole | Tablet | Thiamazole Tablets | 15 | Yes | 15 | 0 |
| | | | Thiamazole Enteric-coated Tablets | 1 | Yes | 1 | 0 |
| 35 | Cyclophosphamide | Injection | Ifosfamide for Injection | 18 | Yes | 16 | -2 |
| | | | Cyclophosphamide for Injection | 5 | Yes | 4 | -1 |
| 36 | Methotrexate | Injection | Methotrexate for Injection | 11 | Yes | 11 | 0 |
| | | | Methotrexate Injection | 2 | Yes | 3 | +1 |
| 37 | Mercaptopurine | Tablet | Mercaptopurine Tablets | 6 | Yes | 7 | +1 |
| 38 | Cytarabine | Injection | Cytarabine Hydrochloride for Injection | 5 | Yes | 5 | 0 |
| | | | Cytarabine for Injection | 2 | Yes | 3 | +1 |
| 39 | Mitoxantrone | Injection | Mitoxantrone Hydrochloride for Injection | 6 | Yes | 6 | 0 |
| | | | Mitoxantrone Hydrochloride Liposome Injection | 1 | Yes | 1 | 0 |
| | | | Mitoxantrone Hydrochloride Injection for Tracing | 1 | Yes | 1 | 0 |
| | | | Mitoxantrone Hydrochloride Injection | 1 | Yes | 2 | +1 |
| | | | Mitoxantrone Hydrochloride Sodium Chloride Injection | 1 | Yes | 1 | 0 |
| 40 | Etoposide | Injection | Etoposide Injection | 13 | Yes | 15 | +2 |
| | | | Etoposide Phosphate for | 2 | Yes | 2 | 0 |

| | | | | | | | |
|---|---|---|---|---|---|---|---|
| | | | Injection | | | | |
| | | | Etoposide for Injection | 1 | Yes | 1 | 0 |
| 41 | Bleomycin A5 (Pingyangmycin) | Injection | Bleomycin A5 Hydrochloride for Injection | 3 | Yes | 3 | 0 |
| 42 | Mitomycin | Injection | Mitomycin for Injection | 4 | Yes | 3 | -1 |
| 43 | Bleomycin | Injection | Bleomycin Hydrochloride for Injection | 2 | Yes | 2 | 0 |
| 44 | Vincristine | Injection | Vincristine Sulfate for Injection | 9 | Yes | 9 | 0 |
| 45 | Tretinoin | Tablet | Tretinoin Tablets | 3 | Yes | 2 | -1 |
| 46 | Calcium Gluconate | Injection | Calcium Gluconate Injection | 51 | Yes | 62 | +11 |
| | | | Calcium Gluconate and Sodium Chloride Injection | 1 | Yes | 6 | +5 |
| 47 | Sodium Thiosulfate | Injection | Sodium Thiosulfate Injection | 3 | Yes | 5 | +2 |
| | | | Sodium Thiosulfate for Injection | 1 | Yes | 1 | 0 |
| 48 | Pralidoxime Chloride | Injection | Pralidoxime Chloride Injection | 7 | Yes | 7 | 0 |
| | | | Compound Pralidoxime Chloride Injection | 1 | Yes | 1 | 0 |
| 49 | Methylthioninium Chloride | Injection | Methylthioninium Chloride Injection | 4 | Yes | 3 | -1 |
| 50 | Naloxone | Injection | Naloxone Hydrochloride Injection | 25 | Yes | 25 | 0 |
| | | | Naloxone Hydrochloride for Injection | 13 | Yes | 13 | 0 |

| | | | | | | | |
|---|---|---|---|---|---|---|---|
| 51 | Acetamide | Injection | Acetamide Injection | 1 | Yes | 1 | 0 |
| 52 | Penicillamine | Tablet | Penicillamine Tablets | 3 | Yes | 3 | 0 |
| 53 | Sodium Dimercaptopropane Sulfonate | Injection | Sodium Dimercaptopropane Sulfonate Injection | 3 | Yes | 3 | 0 |
| 54 | Snake Antivenins | Injection | - | - | No | 0 | - |
| 55 | Oxytocin | Injection | Oxytocin Injection | 19 | Yes | 38 | +19 |
| | | | Carbetocin Injection | 7 | Yes | 9 | +2 |
| | | | Oxytocin for Injection | 2 | Yes | 3 | +1 |
| 56 | Posterior Pituitary | Injection | Posterior Pituitary Injection | 7 | Yes | 9 | +2 |
| 57 | Ethzcridine | Injection | Ethzcridine Lactate Injection | 4 | Yes | 4 | 0 |
| | | | Ethacridine Lactate for Injection | 1 | Yes | 1 | 0 |

**Table S4** Detailed information on shortage medicines for which exclusive production exists

| No. | Generic name | Manufacturer | Indications | Drug specifications | Approval number |
|---|---|---|---|---|---|
| 1 | Compound Pralidoxime Chloride Injection | Beijing Huasu Pharmaceutical Co., Ltd. | Organophosphorus pesticide poisoning. | 2ml:Chlorophosphamide 0.4g,Atropine Sulfate 3mg,Benactyzine Hydrochloride 3mg. | H11022547 |
| 2 | Isoprenaline Sulfate Injection | China Resources Double-Crane Pharmaceutical Co.,Ltd. | Cardiac arrest; infectious shock; cardiogenic shock; complete atrioventricular block. | 2ml:1mg | H11020788 |
| 3 | Ethacridine Lactate for Injection | China Resources Double-Crane Pharmaceutical Co.,Ltd. | Induce labor (childbirth). | 0.1g(Isacridine lactate anhydrous) | H11022105 |
| 4 | Mitoxantrone Hydrochloride Liposome Injection | CSPC Zhongnuo Pharmaceutical (Shijiazhuang) Co., Ltd. | Relapsed/refractory peripheral T-cell lymphoma. | 10ml:10mg (based on $C_{22}H_{28}N_4O_6$) | H20220001 |
| 5 | Clofazimine Soft Capsules | Shanxi Liye Pharmaceutical Co., Ltd. | Infection with sulfone-resistant strains; verrucous leprosy; drug-induced acute leprosy reactions; AIDS complicating atypical mycobacterial infections; erythema nodosum of leprosy. | 50mg | H32021093 |
| 6 | Morphine and Atropine Sulfate Injection | Northeast Pharmaceutical Group Shenyang No.1 Pharmaceutical Co., Ltd | Anesthesia; reflex cardiac arrest during anesthesia. | Morphine Hydrochloride 10mg,Atropine Sulfate 0.5mg | H21021924 |
| 7 | Arginine Glutamate | Liaoning Haisco | Elevated blood ammonia due to chronic | 200ml:20g | H20150032 |

| | | | | | |
|---|---|---|---|---|---|
| | Injection | Pharmaceutical Co., Ltd. | liver disease. | | |
| 8 | Magnesium Sulfate for Injection | Liaoning Pharm-Link Pharmaceutical Co., Ltd. | Eclampsia; preterm labor; gestational hypertension; preeclampsia. | 2.5g | H20051792 |
| 9 | Hydrocortisone Acetate Injection | Shanghai General Pharmacetical Co. Ltd. | Allergic diseases; critical toxic infections; hypopituitarism; inflammatory diseases; adrenocortical insufficiency. | 5ml:0.125g | H31021400 |
| 10 | Recombinant Human Urokinase for Injection | Tasly Pharmaceutical Group Co., Ltd. | Acute ST-segment elevation myocardial infarction. | 5mg(500,000IU)/strip | S20110003 |
| 11 | Neostigmine Methylsulfate for Injection | Jiangsu Jiuxu Pharmaceutical Co., Ltd. | Myasthenia gravis; urinary retention; postoperative functional bowel distension. | 1mg | H20040691 |
| 12 | Ofloxacin and MannitolInjection | Henan Yonghe Pharmaceutical Co., Ltd. | Complicated urinary tract infections; typhoid fever; cervicitis; respiratory tract infections; simple urinary tract infections; gastrointestinal tract infections; infections of the skin and skin structures; lung infections caused by gram - bacteria; bone and joint infections; gonorrheal urethritis; septicemia; acute bronchitis caused by gram - bacteria; bacterial prostatitis; infections of the genitourinary system; infections with sensitive bacteria. | 100ml:Ofloxacin 0.2g with mannitol 5.3g | H10950145 |
| 13 | Human Thrombin for External use,Freeze-dried | Hualan Biological Engineering, Inc. | Postoperative bleeding. | 500IU/1ml/vial | S20050092 |
| 14 | Compound Allopurinol | Guangdong Shixin | Hyperuricemia; gout. | Each tablet contains 100mg of | H20094159 |

| | | | | | |
|---|---|---|---|---|---|
| | Tablets | Pharmaceutical Co., Ltd. | | Allopurinol and 20mg of Benzbromarone. | |
| 15 | Sodium Nitroprusside Injection | Hainan Puli Pharmaceutical Co., Ltd. | Hypertensive crisis; acute congestive heart failure. | 2 ml:50 mg [as $Na_2[Fe(CN)_5NO]$-$2H_2O$] | H20223226 |
| 16 | Allopurinol Sustained-release Tablets | Hainan Puli Pharmaceutical Co., Ltd. | Gout; gout stones; uric acid kidney stones; uric acid nephropathy; hyperuricemia. | 0.25g | H20041743 |
| 17 | Mitoxantrone Hydrochloride Sodium Chloride Injection | Sichuan Meida Kanghuakang Pharmaceutical Co., Ltd. | Prostate Cancer; Colorectal Cancer; Testicular Tumors; Kidney Cancer; Melanoma; Breast Cancer; Ovarian Cancer; Multiple Myeloma; Malignant Lymphoma; Acute Leukemia; Hepatocellular Cancer; Head and Neck Malignancies; Endometrial Cancer; Soft Tissue Sarcoma; Lung Cancer. | 100ml:5mg | H10980248 |
| 18 | Thiamazole Enteric-coated Tablets | Guizhou Shengjitang Pharmaceutical Co., Ltd. | Hyperthyroidism. | 10mg | H20080775 |
| 19 | Etoposide for Injection | Yunnan Botanical Pharmaceutical Co., Ltd. | Neuroblastoma; gastric cancer; rhabdomyosarcoma; small cell lung cancer; malignant lymphoma; malignant germ cell tumor; esophageal cancer; leukemia; non-small cell lung cancer; ovarian cancer. | 40mg | H20060014 |
| 20 | Mepivacaine Hydrochloride and Adrenaline Injection | SEPTODONT | Oral local infiltration anesthesia; dental local infiltration anesthesia. | 1.8ml/strip | HJ20171134 |
| 21 | Verapamil Hydrochloride for Injection | Jilin Jinsheng Pharmaceutical Co. Ltd. | Paroxysmal supraventricular tachycardia; atrial flutter; atrial fibrillation. | 5mg | H20031345 |
| | | | | 10mg | H20031346 |
| 22 | Sodium Thiosulfate for | Shanghai ShangPharma | Iodine poisoning; lead poisoning; cyanide | 0.32g | H31020533 |

| | | | | | |
|---|---|---|---|---|---|
| | Injection | Xinya Pharmaceutical Co. Ltd. | poisoning; arsenic poisoning; mercury poisoning; bismuth poisoning. | 0.64g | H31020534 |
| 23 | Magnesium Sulfate Glucose Injection | Shanghai Changzheng Fumin Jinshan Pharmaceutical Co., Ltd. | Spasms; convulsions; preeclampsia; moderate to severe gestational hypertension; eclampsia. | 100ml: magnesium sulfate 1g, glucose 5g | H20020017 |
| | | | | 250ml: magnesium sulfate 2.5g, glucose 12.5g | H20020018 |
| 24 | Fleroxacin and Mannitol Injection | Jiangsu Inbesi Technology Development Co., Ltd. | Gynecologic infections; Allergenic infections; Respiratory infections; Digestive infections; Soft tissue skin infections; Sepsis; Enterobacteriaceae infections (genus); Genitourinary infections. | 100ml: Floxacin 0.2g, Mannitol 5g | H20020610 |
| | | | | 100ml: Floxacin 0.4g with mannitol 5g | H20000452 |
| 25 | Mitoxantrone Hydrochloride Injection for Tracing | Shanghai Truno Pharmaceutical Co., Ltd. | Lymph node tracing in thyroid cancer. | 0.5ml:2.5mg | H20237085 |
| | | | | 1ml：5mg | H20227061 |
| | | | | 2ml:10mg | H20210025 |
| 26 | Acetamide Injection | Shandong Xinhua Pharmaceutical Co., Ltd. | Sodium fluoroacetate poisoning; fluoroacetamide poisoning; glycofluoride poisoning. | 5ml:2.5g | H37023213 |
| | | | | 2ml:1g | H37023214 |
| | | | | 10ml:5g | H37023215 |

**Table S5** Detailed information on exclusively manufactured varieties of APIs included in monitoring

| No. | API Name | Manufacturer | Registration number | Results of the joint review with durg preparations | Approval number |
|---|---|---|---|---|---|
| 1 | Dibrommannitol | Beijing Saier Biological Pharmaceutical Co., Ltd. | Y20190007437 | A | H11021217 |
| 2 | Corticotrophin | Shanghai ShangPharma No.1 Biochemical Pharmaceutical Co., Ltd. | Y20190002770 | A | H31022942 |
| 3 | Sodium Dimercaptopropane Sulfonate | Shanghai Wanxiang Pharmaceutical Co., Ltd. | Y20220001130 | I | / |
| | | | Y20190001405 | A | H20033618 |
| 4 | Bleomycin Hydrochloride | Zhejiang Hisun Pharmaceutical Co., Ltd. | Y20190001866 | A | H20051232 |
| 5 | Mitoxantrone | Brilliant Pharmaceutical Co. Ltd. | Y20190005755 | A | H10960189 |

Note: Results of the co-review with the durg preparation: A indicates raw materials/excipients/packaging materials that have been approved for use in the listed preparation, and I indicates raw materials/excipients/packaging materials that have not yet passed the co-review and approval with the preparation.
